# Case Report: A novel CIITA mutation causing MHC class II deficiency: first reported case in Morocco

**DOI:** 10.3389/fimmu.2025.1707772

**Published:** 2025-11-25

**Authors:** Aziza Bachir Kattra, Fatima Ailal, Ibtihal Benhsaien, Mohammed Fahi, Asmaa Drissi Bourhanbour, Ahamada Elamine, Zahra Aadam, Abderrahmane Errami, Ahmed Aziz Bousfiha, Jalila El Bakkouri

**Affiliations:** 1 Laboratory of Clinical Immunology, Infection, and Autoimmunity (LICIA), Faculty of Medicine and Pharmacy of Casablanca (FMPC), Hassan II University, Casablanca, Morocco; 2 Department of Pediatric Infectious Diseases and Clinical Immunology, Abderrahim El Harouchi Mother-Child Hospital, University Hospital Center Ibn Rochd, Casablanca, Morocco; 3 Immunology Laboratory, University Hospital Center Ibn Rochd, Casablanca, Morocco; 4 Immunopathology–Immunotherapy–Immunomonitoring Laboratory, Faculty of Medicine, Mohammed VI University of Health and Sciences (UM6SS), Casablanca, Morocco

**Keywords:** major histocompatibility complex class II deficiency, CIITA gene, bare lymphocyte syndrome (BLS), lymphopenia CD4, HLA-DR expression, consanguinity, Morocco

## Abstract

Major histocompatibility complex class II (MHC II) deficiency (bare lymphocyte syndrome type II) is an autosomal-recessive combined immunodeficiency caused by pathogenic variants in the transcriptional regulators CIITA, RFXANK, RFX5, or RFXAP. While RFXANK founder mutations predominate in North Africa, CIITA-related disease is extremely rare. We report two siblings from a consanguineous Moroccan family with the classic early-infancy phenotype. The elder sister developed recurrent febrile rashes, oral candidiasis, and locoregional BCGitis with acid-fast bacilli in granulomas, followed by progressive respiratory failure and fatal cytomegalovirus pneumonitis despite antiviral therapy; immunology showed profound CD4^+^ lymphopenia with CD4/CD8 inversion, near-absent HLA-DR on B cells, undetectable IgG/IgA, and elevated IgM. The proband, identified during family follow-up, had recurrent mucocutaneous infections, marked CD4^+^ lymphopenia with CD4/CD8 inversion, and near-absent HLA-DR on B cells; he was started on monthly intravenous immunoglobulin and trimethoprim–sulfamethoxazole prophylaxis. Targeted next-generation sequencing revealed a novel homozygous nonsense CIITA variant (c.1615C>T; p.R539*), predicted to truncate the GTP-binding domain, abolish downstream leucine-rich repeats, and undergo nonsense-mediated decay, and classified as pathogenic according to ACMG criteria. Molecular confirmation enabled genetic counseling, cascade testing, withholding BCG, and urgent evaluation for allogeneic hematopoietic stem-cell transplantation. This case, likely the first CIITA-related MHC II deficiency reported from Morocco, expands the regional genotypic spectrum and underscores the value of early HLA-DR flow-cytometric assessment and prompt molecular testing in infants from consanguineous families to guide prophylaxis, vaccination decisions, and timely referral for curative therapy.

## Introduction

Major histocompatibility complex class II (MHC II) deficiency, also known as bare lymphocyte syndrome type II, is an autosomal recessive combined immunodeficiency caused by mutations in one of four key transcriptional regulators of MHC-II gene expression: CIITA (group A), RFXANK (group B), RFX5 (group C) or RFXAP (group D) (1–3). Loss of these regulators results in absent or markedly reduced MHC II expression on antigen-presenting cells, leading to defective CD4^+^ T-cell development and activation, as well as impaired T helper-dependent antibody responses (3, 4). Clinically, patients present with profound CD4 lymphopenia, hypogammaglobulinemia, and increased susceptibility to viral, bacterial, mycobacterial, fungal, and protozoal infections (1–4). Without curative hematopoietic stem-cell transplantation (HSCT), most affected children die in early childhood (5, 6).

Worldwide, approximately 200–250 cases have been documented (7–9). The disorder has a pan ethnic distribution but is especially prevalent in North Africa and the Middle East, largely due to a high rate of consanguinity and recurrent founder mutations (3, 8–11). In North African cohorts, nearly all patients belong to complementation group B, caused by a recurrent 26-base pair deletion in *RFXANK* (c.338-25_338del26, also known as 752delG-25) (8, 10–14). This mutation, found in about 90% of North African patients, many of them unrelated, reflects a strong founder effect (3, 8, 10–12, 15). By contrast, *CIITA* (group A) mutations are infrequent globally, accounting for less than 9% of reported MHC class II deficiency cases (16). To date, no CIITA-deficient case has been reported from Morocco. Here, we describe a consanguineous family in which a novel homozygous CIITA nonsense mutation (c.1615C>T; p.R539*) was identified in two siblings. This observation broadens the genotypic spectrum of MHC class II deficiency in North Africa.

## Case presentation

### Patient 1 (proband)

The proband (P1) was a male infant born at term to first-degree consanguineous Moroccan parents. He was the youngest of five siblings; one older brother had died in early infancy of an unknown cause, and an older sister (Patient 2, described below) had died at 14 months of severe pneumonia. Given this family history, a screening immunological workup was performed at 2 weeks of age to rule out an inborn error of immunity. At that time, the patient was clinically asymptomatic.

**Figure 1:**
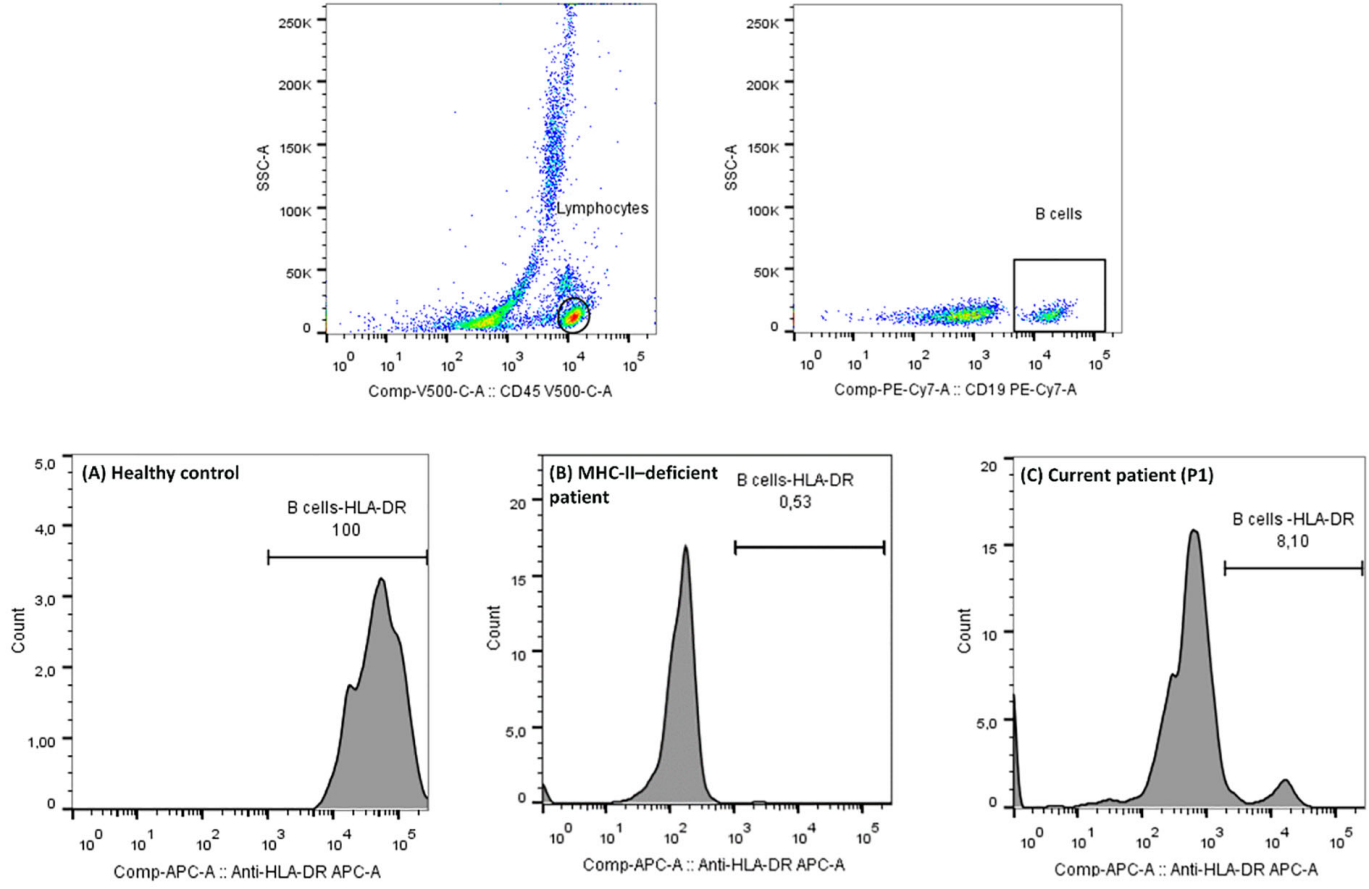
Flow cytometry analysis of HLA-DR expression. Gating strategy (top panels) showing lymphocyte and CD19^+^ B-cell selection, followed by representative histograms of HLA-DR expression (bottom panels). **(A)** Healthy control displaying normal HLA-DR expression (99.4%). **(B)** Patient with complete MHC-II deficiency due to a RFXANK splice-site deletion (c.338-25_338del26) showing an absence of HLA-DR expression (0.53%). **(C)** Current patient (P1) exhibiting markedly reduced HLA-DR expression (8.10%). The positivity threshold in panel C was adjusted to capture a small residual population distinct from the major HLA-DR–negative B-cell population.

Laboratory evaluation showed normal blood counts and serum immunoglobulins of IgG 3.89 g/L, IgA 1.42 g/L, and IgM 0.40 g/L (**Table 1**). These values may reflect residual maternal antibodies. We did not repeat immunoglobulin testing at a later age, as the patient had already been started on intravenous immunoglobulin (IVIG) therapy. Flow cytometry revealed CD4^+^ T-cell lymphopenia (555 cells/mm³), an expansion of CD8^+^ T cells (2430 cells/mm³), and an inverted CD4/CD8 ratio (0.23). B-cell and NK-cell counts were within normal limits. Crucially, HLA-DR expression was reduced on CD19^+^ B cells. The patient exhibited residual (‘leaky’) HLA-DR expression (~8%) (**Figure 1**).

**Table 1:** Comparative paraclinical and immunological assessment of the affected siblings.

Parameter	P1	Reference range*	P2	Reference range*
Hemoglobin (g/dL)	11.9	*12.5–20.5*	8.2	*10.5–13.5*
Leukocytes (cells/mm³)	10,809	*7,200–18,000*	4,700	*6,400–13,000*
Neutrophils (cells/mm³)	3,809	*1,900–5,000*	3,168	*1,000–6,000*
Lymphocytes (cells/mm³)	5,778	*3,400–7,600*	1,424	*3,400–9,000*
Platelets (×10³/µL)	554	*170–500*	—	*200–550*
IgG (g/L)	3.89	*6.5–12.1*	<0.33	*2.4–4.4*
IgA (g/L)	1.42	*0.07–0.22*	<0.06	*0.27–0.86*
IgM (g/L)	0.40	*0.13–0.37*	1.34	*0.34–1.14*
CD3^+^ (cells/mm³)	3,050	*2,500–5,500*	2,596	*1,900–5,900*
CD4^+^ (cells/mm³)	555	*1,600–4,000*	519	*1,400–4,300*
CD8^+^ (cells/mm³)	2,430	*560–1,700*	2,030	*500–1,700*
NK (CD16^+^CD56^+^) (cells/mm³)	405	*170–1,100*	708	*160–950*
CD19^+^ B cells (cells/mm³)	828	*300–2,000*	—	*610–2,600*
CD19^+^HLA-DR^+^ (% of B cells)	8%	*>95%*	11%	*>95% (healthy controls)*

NK, natural killer cells. *Reference ranges: Hematology values were adapted from Hematology reference values by age (CHU Angers – Université d’Angers, 2014; hematocell.fr (17). Immunoglobulin and lymphocyte subset ranges were based on the PID Phenotypical Diagnosis App by Leïla Jeddane et al., 2017, developed from IFCC pediatric reference data and Shearer WT et al., J Allergy Clin Immunol 2003; 112:973–80 (18, 19).

These findings, despite the absence of clinical manifestations at that stage, were diagnostic of MHC II deficiency. The patient was started on monthly IVIG infusions and trimethoprim–sulfamethoxazole prophylaxis as preventive management while awaiting definitive therapy.

### Patient 2 (older sister)

**Figure 2:**
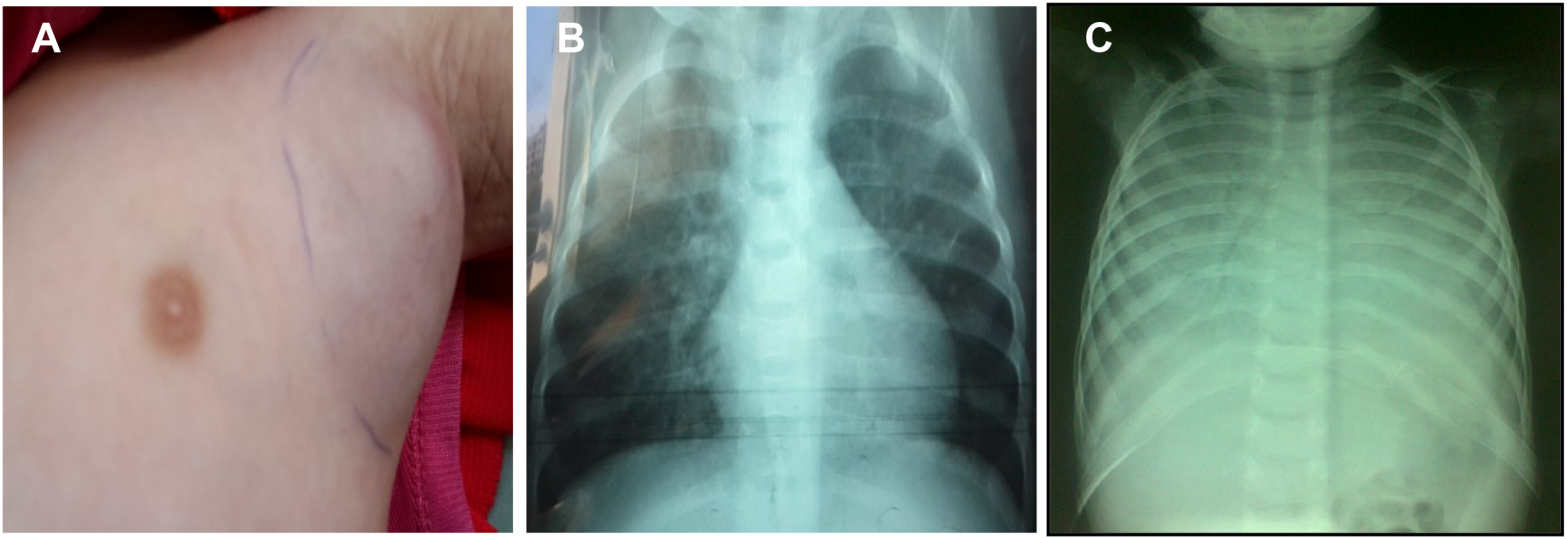
Clinical and radiological findings in Patient 2. **(A)** Left axillary lymphadenopathy consistent with locoregional BCGitis. **(B)** Baseline chest radiograph showing perihilar and bronchial interstitial markings. **(C)** Follow-up chest radiograph demonstrating diffuse bilateral pulmonary opacification consistent with CMV pneumonitis.

Patient 2 (P2), the proband’s older sister, experienced multiple early hospitalizations for febrile skin rashes, oral thrush, and urinary tract infections. At 7 months of age, her weight was 5.2 kg (≈–2 SD) for a normal length of 69 cm. Clinical examination revealed a generalized pustular rash (including perioral and diaper areas), oral candidiasis, a firm and mobile 4-cm left axillary lymphadenopathy (**Figure 2A**) and mild splenomegaly. Lymph node biopsy showed non-caseating epithelioid granulomas containing acid-fast bacilli, while the tuberculin skin test was negative, consistent with locoregional BCGitis. Urine culture grew pan sensitive *Pseudomonas aeruginosa* and chest radiography showed diffuse bronchial infiltrates (**Figure 2B**). HIV testing was negative.

Immunophenotyping revealed CD4^+^ T-cell lymphopenia (519 cells/mm³), whereas other lymphocyte subsets were within normal ranges. HLA-DR expression was markedly reduced, with only 11% of B cells expressing HLA-DR. Serum immunoglobulins showed undetectable IgG and IgA, along with elevated IgM (1.70 g/L; age-matched normal 0.3–0.9 g/L). This profile — profound CD4 lymphopenia and markedly reduced HLA-DR expression — was highly suggestive of MHC class II deficiency. The comparative paraclinical and immunological data of both siblings are summarized in **Table 1**.

She was treated with anti-tubercular therapy (for presumed BCGitis), monthly IVIG, and trimethoprim-sulfamethoxazole prophylaxis. Initially, her fever and lymphadenopathy regressed. Two months later, she developed high fever (38.5 °C), tachypnea, and respiratory distress. Chest radiography then showed bilateral “white-out” lung fields consistent with interstitial pneumonia (**Figure 2C**). Blood PCR for cytomegalovirus (CMV) was strongly positive (>210,000 IU/mL; threshold <350 IU/mL). Despite treatment with ganciclovir followed by foscarnet, her condition deteriorated rapidly, and she died 12 days after admission due to CMV pneumonitis and respiratory failure. Her clinical course and immunological profile were highly consistent with MHC II deficiency, although molecular confirmation could not be obtained because no post-mortem samples were available.

## Genetic analysis

**Figure 3:**
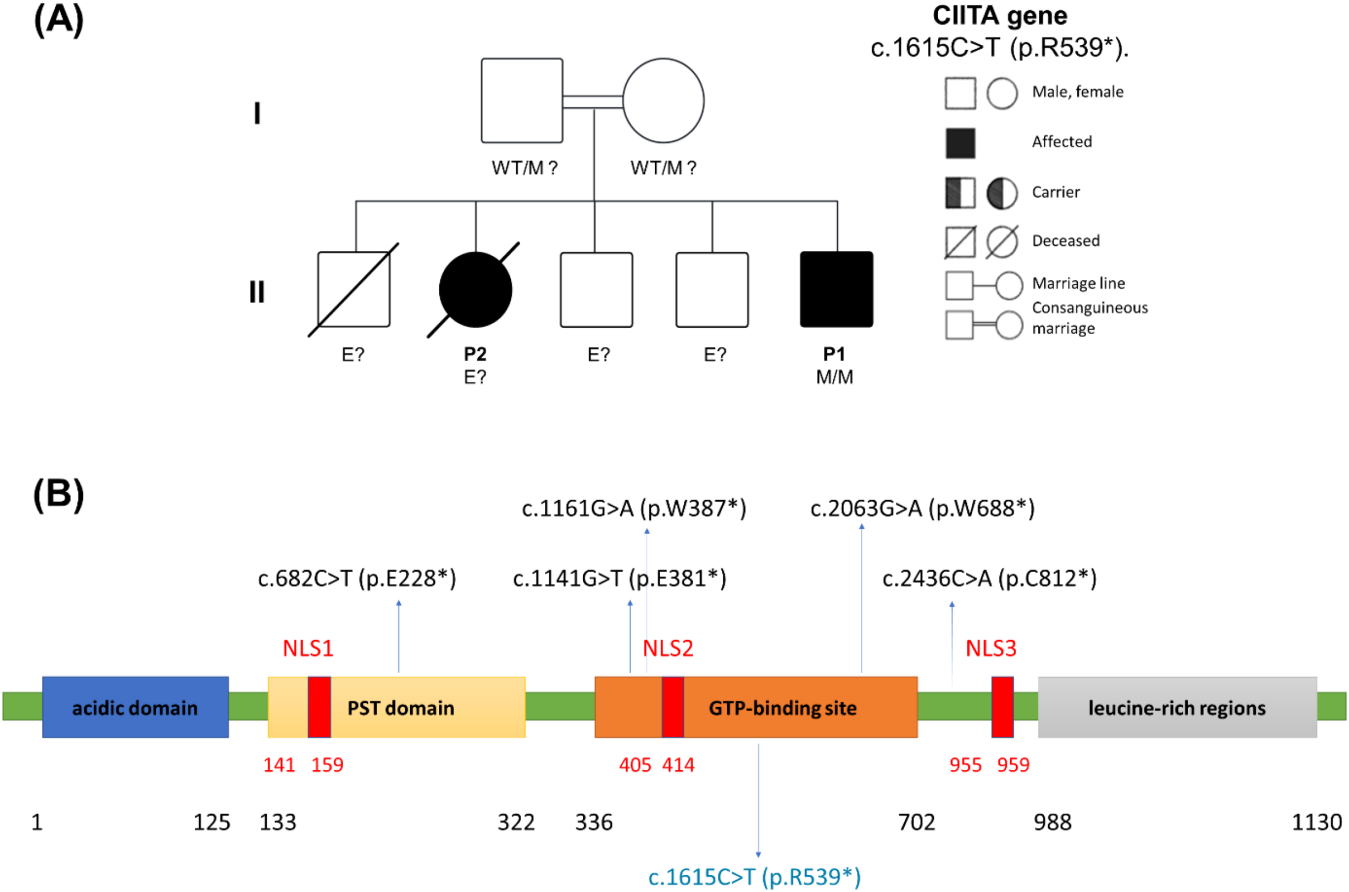
Genetic findings and CIITA domain organization. **(A)** Pedigree of the affected consanguineous Moroccan family with the *CIITA* c.1615C>T (p.R539*) variant, showing autosomal-recessive inheritance. P1: homozygous mutant (M/M); P2: clinically affected, not genetically tested; parents: presumed heterozygous carriers (WT/M)?; E?: not evaluated; WT, wild type; M, mutant allele. **(B)** Schematic representation of the CIITA protein (1–1130 aa) illustrating the acidic activation domain (1–125), the proline-serine-threonine (PST) domain (125–336), the GTP-binding region (336–702), and the leucine-rich regions (702–1130). Nuclear localization signals (NLS1–3) are shown in red (141–159, 405–414, 955–959). Previously reported nonsense variants (p.E228*, p.E381*, p.W387*, p.W688*, p.C812*) are indicated by arrows, and the novel family variant c.1615C>T (p.R539*) is highlighted in blue within the GTP-binding region.

Targeted next-generation sequencing of P1 using a primary immunodeficiency panel identified a novel homozygous nonsense variant in CIITA (NM_000246.4), c.1615C>T (p.R539*), located within exon 11. Both parents were healthy and consanguineous, supporting autosomal-recessive inheritance within the pedigree (**Figure 3A**). CIITA encodes the non–DNA-binding master regulator of MHC class II transcription, and loss-of-function variants are a known cause of MHC II deficiency.

The p.R539* substitution introduces a premature termination codon within the GTP-binding domain (amino acids 336–702), resulting in truncation before the leucine-rich repeat region required for CIITA–coactivator interactions and nuclear localization (**Figure 3B**). Because the stop codon occurs upstream of the final exon–exon junction, nonsense-mediated mRNA decay is predicted, leading to a null allele and complete loss of CIITA function.

**Figure 4:**
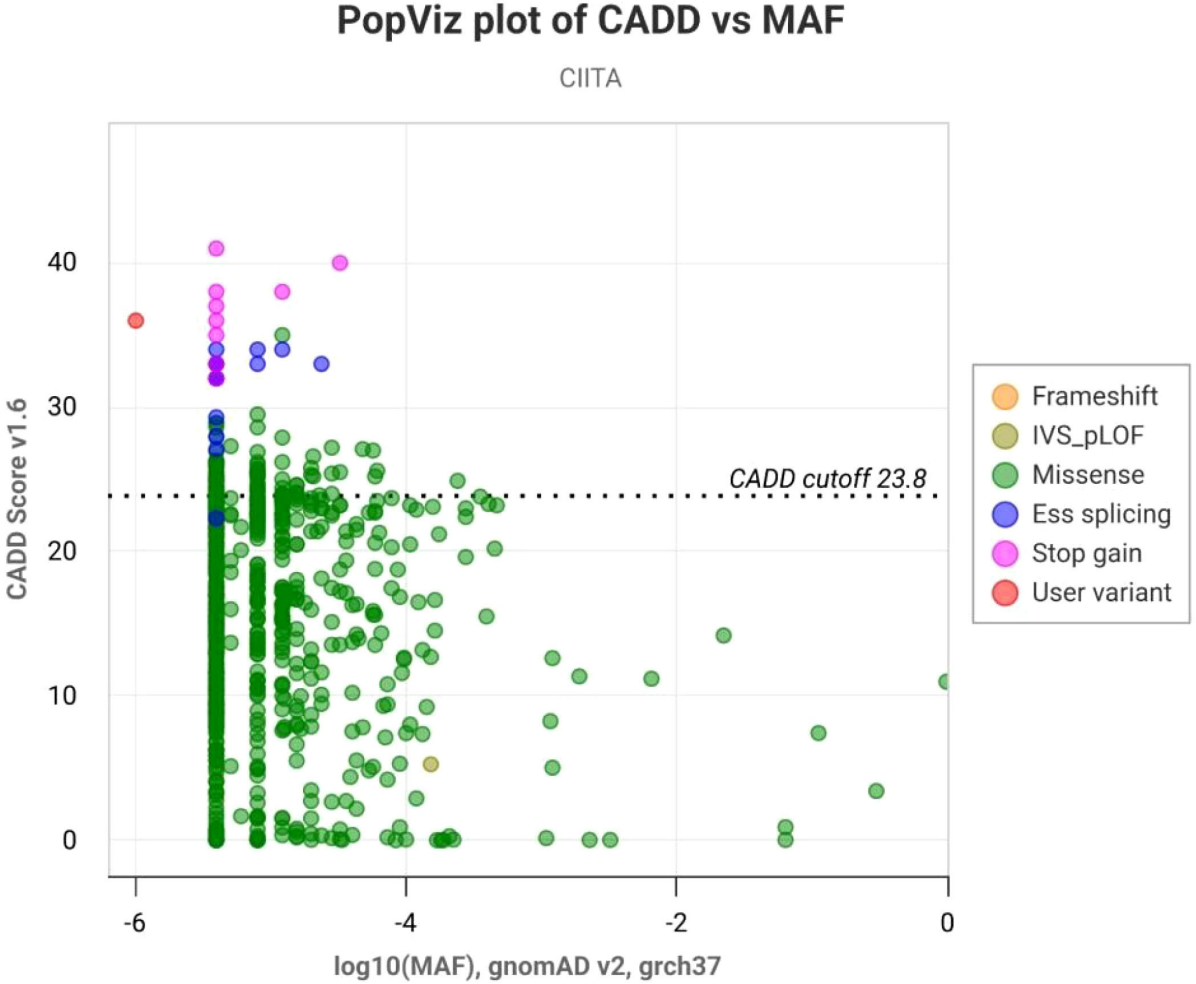
PopViz analysis of the CIITA c.1615C>T (p.R539*) variant. PopViz plot showing the relationship between Combined Annotation-Dependent Depletion (CADD) scores (y-axis) and Minor Allele Frequency (MAF, x-axis, log10 scale) for variants in CIITA (gnomAD v2, GRCh37). Each dot represents a reported CIITA variant, color-coded by consequence (frameshift, missense, essential splicing, stop gain, etc.). The dotted line indicates the CADD pathogenicity cutoff (23.8). The novel variant c.1615C>T (p.R539*) is shown as a red dot (MAF ≈ 10^-6^, CADD score = 36), positioning it well above the pathogenicity threshold and supporting its predicted deleterious effect.

The variant was absent in the homozygous state and extremely rare overall in gnomAD, further supporting its pathogenicity. In silico prediction tools (BayesDel, CADD = 36, PopViz) consistently indicated a deleterious impact (**Figure 4**), and the variant is classified as pathogenic in ClinVar (**Supplementary Table S1**). Applying ACMG/AMP criteria, the variant fulfills PVS1 (null variant in a gene with established loss-of-function mechanism), PM2 (absent from controls), and PP4 (highly specific phenotype of MHC II deficiency), confirming its classification as pathogenic.

Genetic counseling was provided, and P1 remains on monthly immunoglobulin replacement while undergoing evaluation for allogeneic HSCT, the only curative treatment currently available.

## Discussion

This report documents the first Moroccan cases of major histocompatibility complex (MHC) class II immunodeficiency due to a novel biallelic CIITA variant in two siblings born to consanguineous parents. The immunological phenotype was consistent and highly suggestive of this diagnosis, showing markedly reduced HLA-DR expression, profound CD4^+^ T-cell lymphopenia, and impaired immunoglobulin production. Clinically, the older sister presented from early infancy with the classic phenotype of MHC II deficiency, including severe recurrent infections and failure to thrive (1–4). In particular, she had received BCG vaccination, developed locoregional BCGitis, and ultimately died of cytomegalovirus (CMV) pneumonia, highlighting the profound impairment of CD4^+^-mediated activation and antiviral responses in the absence of MHC II. By contrast, the proband (P1), screened early because of the family history, was asymptomatic and had not received BCG vaccination. Although severe BCG disease is uncommon, disseminated BCGitis has been reported, including a probable pre-transplant case from Iran and a post-transplant case (20, 21). Severe viral infections, notably CMV, are a well-recognized cause of morbidity and mortality in MHC II deficiency (10).

Immunologically, both siblings showed residual HLA-DR expression (~8–12%). P1 had hypogammaglobulinemia, whereas his sister (P2) presented absent IgG/IgA with elevated IgM, an “hyper‐IgM‐like” serology. Cohort data indicate that a minority of patients exhibit elevated IgM despite global T-helper dysfunction (e.g., 9% in a North-African RFXANK-deficient series) (10), and variable immunoglobulin levels (low, normal, or high) have been reported elsewhere (13). The residual HLA-DR expression seen in P2 may represent a “leaky” phenotype, as reported in certain CIITA mutations, which can permit partial activity and variable clinical severity (10, 22). Finally, our observations align with reports that patients with MHC II deficiency may retain detectable T-cell receptor excision circles (TRECs) despite profound CD4^+^ lymphopenia, reflecting that early T-cell development remains intact, but positive selection and expansion fail in the absence of MHC II (3). This underscores why TREC-based newborn screening may fail to identify such cases (23, 24). Collectively, these data reinforce the diagnostic value of early immune phenotyping, especially flow cytometry for lymphocyte subsets and HLA-DR expression, in infants from consanguineous families presenting with severe, recurrent infections and failure to thrive.

Genetically, MHC II deficiency is heterogeneous, with pathogenic variants not in HLA structural genes but in four transcriptional regulators: class II transactivator CIITA (group A) and the RFX complex subunits RFXANK (group B), RFX5 (group C), and RFXAP (group D) (3). CIITA mutations are the least common cause worldwide, while RFXANK deficiency accounts for >70% of all patients and in ~90% of North African cohorts (3, 10). In Maghreb populations, the 26-bp founder deletion in RFXANK accounts for nearly all reported cases (8, 10–14). By contrast, CIITA deficiency is exceptionally rare in this region, with only a handful of mutations described, mostly in Europe and the Middle East (8, 25, 26). No Moroccan patient with CIITA deficiency had been reported before this study; thus, this family represents the first Moroccan cases and expands the genotypic spectrum in Morocco and North Africa. A recent review identified only ~18 CIITA-deficient patients worldwide, mostly with missense, nonsense, or splicing variants. The novel CIITA variant described here (c.1615C>T; p.R539*) has not been reported previously. Mechanistically, it introduces a premature stop codon in the GTP-binding domain, abolishing the downstream leucine-rich repeat region required for nuclear import and transcriptional activation, and is predicted to undergo nonsense-mediated decay, yielding a functional null allele. The siblings were homozygous for the variant, and both parents were obligate heterozygous carriers, confirming autosomal-recessive inheritance. According to ACMG/AMP criteria, this stop-gain meets PVS1, PM2, and PP4, and is therefore classified as pathogenic.

Most pathogenic CIITA mutations reported to date are truncating or inactivating, consistent with the essential role of CIITA in MHC class II expression (26, 27). Previously reported nonsense variants include p.E228*, p.E381*, p.W387*, p.W688*, and p.C812*, with clinical–immunological features converging toward infancy-onset severe infections, near-complete loss of HLA-DR on APCs, marked CD4^+^ lymphopenia, and low IgG/IgA (IgM variably preserved) (23, 28–30). Our two Moroccan siblings (homozygous p.R539*) closely fit this archetypal pattern of early-onset, complete MHC II deficiency. These truncating alleles abrogate CIITA function by eliminating the C-terminal LRR domain, underscoring their severe loss-of-function effect. By contrast, missense CIITA variants can show a broader phenotypic range, from severe early-onset disease to relatively milder or later-presenting courses, including “leaky” immunological phenotypes (22, 25, 31). Nonetheless, even “leaky” CIITA defects ultimately result in significant immunodeficiency, and nearly all patients, whether harboring nonsense or missense variants, require aggressive supportive therapy and early consideration of curative hematopoietic stem-cell transplantation (HSCT) (32, 33).

Genetic testing provided definitive confirmation of MHC II deficiency in this family and has immediate clinical utility: it enables targeted counseling of parents and siblings, supports cascade carrier testing, and offers the possibility of prenatal diagnosis in future pregnancies, in line with recent reports on CIITA and the RFX complex (27, 34, 35). The proband was identified during family follow-up and was not vaccinated with live BCG, preventing complications. Beyond this family, documenting this variant expands the mutational spectrum of CIITA deficiency and will facilitate recognition in future cases and potential inclusion in variant databases.

Therapeutically, MHC II deficiency remains a major challenge. Allogeneic HSCT is the only curative treatment, but outcomes have historically been poor due to pre-existing infections, conditioning toxicity, graft rejection, and GVHD, as repeatedly noted in aggregated series (33, 35, 36). More recent data suggest improved survival with optimized supportive care, reduced-toxicity conditioning, refined serotherapy/T-cell depletion, and vigilant anti-infective strategies, though many children still die young without transplantation (33). In resource-limited settings like ours, these hurdles are magnified: diagnosis and referral are often delayed, active infections are frequent, and few centers have transplant expertise in primary immunodeficiencies. In line with current best practice, our patient is receiving antimicrobial prophylaxis and monthly IVIG while awaiting HSCT. However, such supportive care is only palliative; without HSCT the prognosis remains poor. Access to HSCT will be critical but must be carefully timed given the risks (32, 33).

This report has several limitations. First, although the strong concordance between the genotype and immunophenotype supports a loss-of-function effect of the CIITA variant, we did not perform functional validation experiments such as mRNA or protein quantification, nuclear localization, or rescue assays, which would have provided direct mechanistic evidence. Second, functional lymphocyte assays, including antigen- or mitogen-induced proliferation, cytokine production, or HLA-DR induction, were not available, limiting the assessment of cellular immune competence. Third, Sanger confirmation and extended segregation analysis were not performed, preventing verification of carrier status in the wider family. Fourth, although flow cytometry provided diagnostic confirmation through markedly reduced HLA-DR expression, expression on monocytes was not measured, and isotype controls were not included, in accordance with routine practices in clinical immunology laboratories; nonetheless, their inclusion would have added technical rigor and allowed finer interpretation of the residual HLA-DR signal observed. In addition, the original flow-cytometry plots for P2 were unavailable, as the patient died years earlier and the data were retrieved from archived medical records, precluding direct side-by-side comparison with her sibling. Finally, this report describes a single consanguineous family, and whole-exome sequencing, while diagnostic, cannot exclude non-coding or structural variants that might modify disease expression.

## Conclusion

In summary, we describe a novel CIITA nonsense mutation (c.1615C>T; p.R539*) causing MHC class II deficiency in two siblings from a consanguineous Moroccan family. This is the first report of CIITA deficiency in Morocco, and the variant itself is reported here for the first time worldwide, expanding the genetic spectrum of bare lymphocyte syndrome beyond the well-known North African founder mutation in RFXANK. Clinically, our findings emphasize the need to consider MHC II deficiency in infants with recurrent infections, profound CD4 lymphopenia, and reduced HLA-DR expression, particularly in consanguineous families or those with affected siblings. Early immunophenotyping with HLA-DR assessment, followed by timely molecular confirmation, is critical to guide infection prophylaxis, vaccination decisions, and genetic counseling. Prompt recognition also facilitates referral for allogeneic HSCT, the only curative treatment. Finally, in resource-limited settings, increasing clinician awareness and improving access to specialized immunology and transplant services remain essential to reduce diagnostic delays and improve outcomes.

## Data Availability

All data supporting the conclusions of this article are included within the manuscript. No additional datasets were generated.

## Supplementary material

Supplementary Table 1 *In silico prediction scores and ACMG/AMP classification of the CIITA c.1615C>T (p.R539**) variant. The table summarizes pathogenicity predictions from multiple algorithms (BayesDel_addAF, BayesDel_noAF, CADD, PopViz), allele frequency data from gnomAD, ClinVar classification, and the final ACMG/AMP interpretation (PVS1, PM2, PP4 → Pathogenic). BayesDel_addAF and BayesDel_noAF are deleteriousness meta-scores; higher values indicate increased pathogenicity (ranges: –1.29 to 0.75 and –1.31 to 0.84, respectively). In this case, BayesDel scores were within the normal range, which is expected for nonsense variants where the deleterious effect is primarily determined by premature truncation and nonsense-mediated decay. By contrast, the very high CADD score (36), the absence of the variant in gnomAD, and its classification as pathogenic in ClinVar support a pathogenic effect. According to ACMG/AMP standards, the variant is therefore classified as Pathogenic.
