# Supplementary material for: Case Report: A novel CIITA mutation causing MHC class II deficiency: first reported case in Morocco
Source: Front Immunol. 2025 Nov 25;16:1707772. doi: 10.3389/fimmu.2025.1707772 (PMC12685675; doi:10.3389/fimmu.2025.1707772)
Supplement: Supplementary Table 1 — In silico prediction scores and ACMG/AMP classification of the CIITA c.1615C>T (p.R539*) variant. The table summarizes pathogenicity predictions from multiple algorithms (BayesDel_addAF, BayesDel_noAF, CADD, PopViz), allele frequency data from gnomAD, ClinVar classification, and the final ACMG/AMP interpretation (PVS1, PM2, PP4 → Pathogenic). BayesDel_addAF and BayesDel_noAF are deleteriousness meta-scores; higher values indicate increased pathogenicity (ranges: –1.29 to 0.75 and –1.31 to 0.84, respectively). In this case, BayesDel scores were within the normal range, which is expected for nonsense variants where the deleterious effect is primarily determined by premature truncation and nonsense-mediated decay. By contrast, the very high CADD score (36), the absence of the variant in gnomAD, and its classification as pathogenic in ClinVar support a pathogenic effect. According to ACMG/AMP standards, the variant is therefore classified as Pathogenic. [file Table1.pdf]

Supplementary Table S1: *In silico prediction scores and ACMG/AMP classification of the CIITA c.1615C>T (p.R539*)* variant.

Predictive tool	Score	Calibrated prediction
**BayesDel_addAF**	0.58	Pathogenic
**BayesDel_noAF**	0.28	Pathogenic
**CADD**	36	Pathogenic

The table summarizes pathogenicity predictions from multiple algorithms (BayesDel_addAF, BayesDel_noAF, CADD, PopViz), allele frequency data from gnomAD, ClinVar classification, and the final ACMG/AMP interpretation (PVS1, PM2, PP4 → Pathogenic).

Notes: BayesDel_addAF and BayesDel_noAF are deleteriousness meta-scores; higher values indicate increased pathogenicity (ranges: –1.29 to 0.75 and –1.31 to 0.84, respectively). In this case, BayesDel scores were within the normal range, which is expected for nonsense variants where the deleterious effect is primarily determined by premature truncation and nonsense-mediated decay. By contrast, the very high CADD score (36), the absence of the variant in gnomAD, and its classification as pathogenic in ClinVar support a pathogenic effect. According to ACMG/AMP standards, the variant is therefore classified as Pathogenic.
